# Valorization of Coffee Silverskin Using Extraction Cycles and Water as a Solvent: Design of Process

**DOI:** 10.3390/molecules29061318

**Published:** 2024-03-15

**Authors:** Aziadé Chemat, Didier Touraud, Rainer Müller, Werner Kunz, Anne-Sylvie Fabiano-Tixier

**Affiliations:** 1 INRAE, UMR 408, Avignon University, F-84000 Avignon, France; 2 Institute of Physical and Theoretical Chemistry, University of Regensburg, 93040 Regensburg, Germany; didier.touraud@chemie.uni-regensburg.de (D.T.); rainer.mueller@chemie.uni-regensburg.de (R.M.); werner.kunz@chemie.uni-regensburg.de (W.K.)

**Keywords:** coffee, silverskin, extraction, water, cycles, enrichment

## Abstract

Coffee silverskin is a byproduct of the coffee industry, appearing in large quantities during the roasting step. In this work, a sober and simple water process is proposed, using extractions cycles, to produce valuable products including (a) an extract rich in caffeine, (b) possibly pure caffeine, and (c) insoluble fibers. The hypothetical number of necessary cycles was calculated and compared to the number of cycles used experimentally. Two types of cycles, with and without water compensation, were compared for their water consumption and the amount of caffeine extracted. The use of cycles, with the resulting product from a previous extraction as a solvent for fresh biomass, drove a significant rise in the content of caffeine determined by a UV–visible detector with a spectrophotometer and ultra-performance liquid chromatography (UPLC). After 11 extraction cycles with water compensation, we obtained an extract 4.5 times more concentrated in caffeine (4.25 mg/mL) than after a single extraction (1.03 mg/mL).

## 1. Introduction

**Figure 1:**
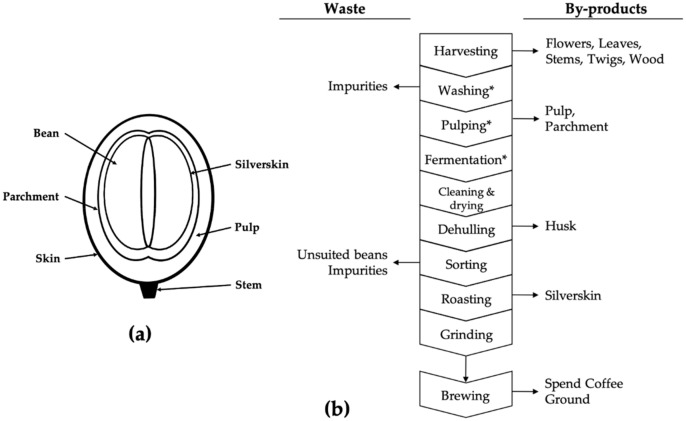
(**a**) Representation of the coffee cherry and its different layers. (**b**) Diagram of wet and dry processing of coffee, and the generated byproducts (* the steps not included in the dry processing). Adapted from the reported information in the literature [1,3,7,8,9,10,11].

*Coffea* sp., also known as coffee, is a plant from the family Rubiaceae, comprising more than 70 varieties [1]. The two most popular of these varieties are *Coffea arabica* (Arabica) and *Coffee canephora* (Robusta), accounting for 75% and 24% of the production, respectively [1]. The fruit of the coffee tree is called the coffee cherry or berry, with an average size of 10 mm, and it contains multiple layers, as shown in Figure 1a. The well-known coffee beans for coffee making are the seeds of the fruit, situated at the center of those layers [1,2,3].

According to recent data, the world’s annual coffee consumption is around 10 million tons. The main producing and exporting countries of coffee are Brazil, Vietnam, and Indonesia, while the main importing and/or consuming regions are the United States of America, Brazil, and Japan [4,5]. The coffee trade places second in the ranking of traded goods, just after the petroleum trade [1,3].

There are two main ways to process the coffee cherry into a bean fit for making coffee, namely, the dry and wet methods, shown in Figure 1b. Multiple byproducts are generated at different stages of this process: flowers, leaves, twigs, wood, and stems during the harvesting, the pulp during the pulping step, the husk during the dehulling, and the silverskin while roasting. Another waste product generated during coffee making is the spent coffee ground [1,6,7]. Coffee is considered to be a significant waste generator due to its form, with approximately 30 to 50% of the total weight of processed coffee being discarded [1,3].

The typical method for the disposal of this waste is to incinerate or discard it, which releases caffeine and CO_2_ into the environment, causing serious environmental issues [1,12]. Due to the recent growing interest in sustainability and environmentally friendly actions, numerous researchers and producers are investing in developing novel approaches to valorize coffee-making-related byproducts. The latter copious waste can be used for animal feed [1,3,8], developing new materials [1,3,12], producing biofuels [1,3,6,8], formulating natural aromas [7,8], or isolating active compounds, which can further be used as ingredients for the food, cosmetic, nutraceutical, or pharmaceutical industries [1,7,13]. Such practices can significantly contribute to increasing the profitability of the coffee industry and establish the concept of a circular economy. This zero-waste approach could benefit producers, stakeholders, and consumers, considering the recent and rapidly growing concerns about industrial pollution [7,14].

Coffee silverskin is an integument around the coffee bean that is separated from the green coffee bean during the torrefaction process, as illustrated in Figure 1a. It is estimated that for every 1 ton of coffee beans being roasted, 7.5 kg of coffee silverskin is separated and discarded [15]. The coffee silverskin is the only industrial byproduct from coffee processing to appear in the consuming country, during the roasting step. Nevertheless, less scientific work has been devoted to the valorization of this byproduct compared to other coffee-derived waste. Several studies have revealed the potential use of this biomass as a functional ingredient or a cosmetic ingredient [16], as a flavoring agent [6,7], as a substrate for insects’ growth and production [17], and as different types of materials [18,19,20]. Coffee silverskin is currently under examination by the authorities for its functional ingredient/novel food status [21,22,23]. Multiple studies have also been conducted to determine the benefits and the safety of this product for human consumption [24,25,26,27]. Epidemiological research studies have already associated the bioactive fraction of coffee silverskin with antioxidative, prebiotic, neuroprotective, and antimicrobial activities [1,13,15,24,26].

**Figure 2:**
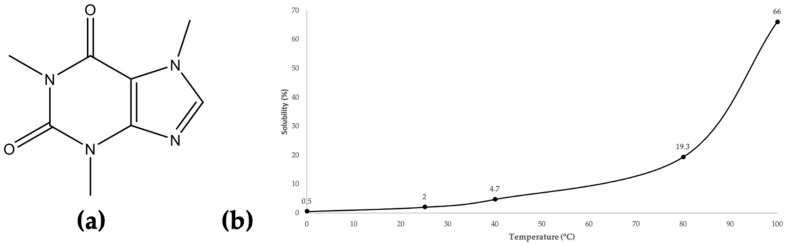
(**a**) Caffeine’s molecular structure. (**b**) Caffeine’s water solubility (*w*/*w*) plotted versus temperature (°C), adapted from the data found in the literature [28,29,30].

Caffeine, which belongs to the xanthine family, is considered to be one of the most valuable compounds in coffee silverskin (see Figure 2a). The solubility versus temperature curve for this compound, derived from data in the literature, is reported in Figure 2b [28,29,30]. This solubility curve is exponential, with an important increase over 70 °C. Caffeine can be found in other plants, such as tea leaves, cocoa beans, guarana seeds, etc. It can be added as an ingredient in energy drinks, pastries, ice creams, candies, or pharmaceuticals [31,32]. Several studies have been published to unveil the effects of caffeine consumption, both natural and synthetic, on the human body. Different health claims are available about caffeine; therefore, it can be considered to be a nutritional ingredient [32].

Keeping this in mind, numerous studies have been conducted for the extraction of health-related compounds from coffee silverskin. For instance, ultrasound-assisted extraction was explored for the recovery of caffeine using a hydroalcoholic mixture [33], water [34], or deep eutectic solvents (DESs) [35]. Supercritical CO_2_ extraction was also investigated to valorize coffee silverskin [34]. Likewise, subcritical water extraction has shown interesting results in the extraction of compounds of interest [6,34]. Using maceration for the extraction of coffee silverskin remains one of the most applied methods, using different solvents, such as acidified water [6,26], an acid-catalyzed hydroalcoholic solution [36], or water. Indeed, many studies have been conducted on the combination of water as a solvent with advanced extraction technologies, including ultrasound- or microwave-assisted extraction and subcritical water extraction, or with conventional maceration. However, the main protocol used for the water-based extraction of coffee silverskin relies on the patent (WO 2013/004873) developed by Castillo et al. [37]. Its principle is based on a one-unit maceration process using boiling water. The reported studies conducted on this patent, and on other water-based maceration procedures, suggest that the resulting aqueous extracts are safe, non-toxic, and suitable as food ingredients [6,24,27,37,38,39].

Hence, the present study aims to valorize coffee silverskin through the extraction of one of its most valuable compounds, caffeine, by establishing a water-based process using extraction cycles to maximize its concentration in the final product. Keeping in mind the fact that water is considered to be an efficient, green, and sustainable solvent [40], this process seems to play a crucial role in reducing agro-industrial waste while minimizing the consumption of both water and energy. To the best of our knowledge, this is the first study exploring the use of multi-cycle extraction of caffeine from coffee silverskin. This will further promote the industrial exploitation of the latter copious waste produced by coffee-related industries for preparing novel natural ingredients and materials, or for other innovative applications.

## 2. Results and Discussion

### 2.1. Extraction and Selection of Parameter Settings

The extraction temperature was fixed at 60 °C beforehand, by following the caffeine solubility curve in Figure 2b. Coffee silverskin containing a caffeine content between 1.25% and 3.7%, a temperature of 40 °C with a 4.7% solubilization should be sufficient. Nevertheless, this temperature is not sufficient to inhibit the formation of limescale and tartar [41] and prevent any enzymatic fermentation due to the possible presence of microorganisms [42] that can be found in the silverskin itself, such as *Aspergillus ochraceus* and *Penicillium verrucosum*, or in water, such as *Legionella pneumophila*. Therefore, a temperature of 60 °C was chosen, since this is beyond the growth and survival temperature range of the abovementioned microorganisms [24,43]. Higher temperatures were avoided, as one of the main goals of the present study was to reduce energy costs. At 60 °C, the solubilization is about 9 wt%, whereas at room temperature it is only 2.0 wt% (see Figure 2b). Nevertheless, in the final extract solution, the caffeine content should be less than 2% to avoid precipitation during storage at room temperature.

Three ratios were chosen, considering the amount of biomass for the lowest ratio and the amount of water used for the second. Since the biomass used in this study was lightweight, a ratio of 1/25 was set as the minimum, while the highest ratio was set to 1/100 to guarantee the cost-effectiveness of the process.

The kinetics experiments were conducted for 30 min, with samplings at 0, 15, and 30 min. The maximal time of 30 min was decided by keeping in mind that the study’s aim was to develop a valorization process that is easily scalable to the industrial level. Extended extraction times could limit the scalability and require more heating time, thus leading to more energy consumption.

**Figure 3:**
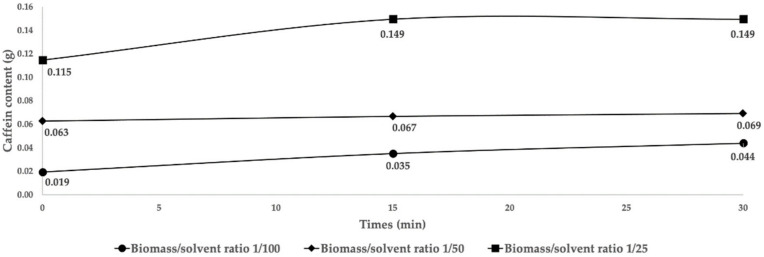
Maceration time effects for different water/silverskin ratios (*w/w*) versus extracted caffeine in 200 mL of water at 60 °C.

In Figure 3, the extracted caffeine contents at the three selected ratios over time are depicted. The considerable amount of this compound at the beginning of the extraction reveals its high solubility in water. In the case of the 1/100 and 1/25 ratios, it can be noted that the contents of caffeine during the 30 min extraction time resulted in an increased difference of 0.025 g and 0.034 g, respectively, while at the 1/50 ratio the increase was only around 0.006 g. There were clear differences in extracted contents between the ratios, with the 1/25 ratio having a caffeine content 3.4 times greater than that of the 1/100 ratio and 2.2 times greater than that of the 1/50 ratio. Therefore, a solid-to-liquid ratio of 1/25 was chosen as optimal for recovering caffeine while also allowing the use of more raw materials and less extraction solvent. Furthermore, 15 min was chosen as the optimal extraction time since no difference was noticed between 15 and 30 min for the selected ratio.

### 2.2. Extraction Cycles and Limiting Parameters

For better recovery of caffeine, an extraction based on multiple cycles holds tremendous interest. As part of this procedure, a cycle is carried out by using the extract of the prior extraction step as the solvent for the following one. This method allows for the continuous enrichment of the extract, the use of more byproducts, and less water consumption.

In this work, the extraction cycle without water addition was analyzed using a UV–visible spectrophotometer, while UPLC coupled with a UV–visible detector was also used for the analysis of the extraction with water compensation. However, even when both analysis methods used UV–visible detectors, differences in the caffeine content could be noted when comparing the results.

The accuracy is not the principal source of deviation between the two methods; as the analyses conducted with a spectrophotometer were performed in water, two effects could have had an impact: The first is due to the formation of a heterocomplex of caffeine and polyphenols [44], leading to a diminution of the absorption value. The second comes from a possible overlap of the absorption curves of other polyphenols with that of caffeine [45,46], leading to an augmentation of the absorption value.

**Figure 4:**
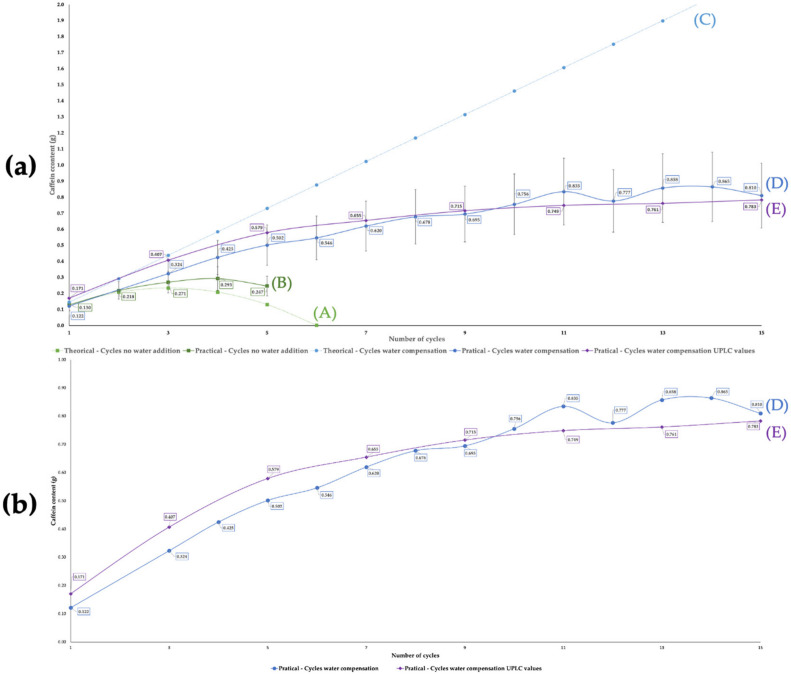
(**a**) Model and experimental curves of caffeine extracted in 200 mL of water with no water (solvation) added (curves (A) and (B)), and with water compensation (curves (C) and (D)), plotted versus the number of extraction cycles at 60 °C using a UV–visible spectrophotometer for the determination of the quantities of caffeine. (**b**) Experimental curve of caffeine extracted with water compensation plotted versus the number of extraction cycles using a spectrophotometer and UPLC (curves (D) and (E)) for the determination of the quantities of caffeine plotted versus the number of extraction cycles at 60 °C.

By comparing curves (D) and (E) in Figure 4b, a low content deviation can be noticed between those measured by spectrophotometer, slightly higher after the 8th cycle, and by UPLC. Therefore, it appears that at low concentrations, deviations due to formation of heterocomplexes are dominant, while at high concentrations they are mainly due to curves overlapping. At intermediate concentrations, an alignment of the values can be observed, suggesting a balance between the two effects.

Comparing curves (D) and (E) in Figure 4b allows us to show that UV–visible measurements conducted via spectrophotometer are adequate as a method to monitor the increase in caffeine content throughout the process. In this case, an error range of 10% was estimated for the data obtained from the spectrophotometer analysis.

Even though UPLC-based methods are known to be more accurate, the use of the spectrophotometer method for observing the increase in caffeine content during the process helps to obtain immediate results and is easier to set up. Therefore, measurements using this spectroscopic method were preferred in the attempt to follow our first goal, designing a process that can be efficient and easy to perform at an industrial scale.

In Figure 4a, curves (A) and (B) are the theoretical and experimental curves, respectively, and represent the quantity of caffeine extracted versus the number of extraction cycles carried out without compensation of the water lost. The comparison of these two curves shows the theoretical model to achieve a good prediction of the process. The model predicts a decrease in the total quantity of extract after six cycles, and the experiment shows a plateau after four cycles. The volume of water during these experiments dropped after each cycle (see Table 1), with an average of 31 mL per cycle, from an initial volume of 200 mL to a final extract of 43 mL. In parallel, the caffeine content rose until reaching a maximum of 0.293 g at the fourth cycle and 0.247 g after the fifth one. Throughout this process, 40.1 g of raw material and 200.6 mL of water were used. The loss of water appears to be a limiting factor to the efficiency of the process, leading to a limited amount of extract.

To counteract the limitation from the water loss due to fiber hydration, in a second process, the water was compensated in each extraction cycle. Here, the losses did not further limit the theoretical number of cycles. The new parameter limiting the calculation was the caffeine’s solubility at 25 °C, this temperature being the one used for the filtration and storage of the extract. At this temperature, the solubility is around 2% (*w*/*w*), meaning the maximum caffeine content should be 4 g for a 200 mL solution. It was calculated that a total of 32 cycles would theoretically be necessary to attain this amount. as shown in Figure 4a (curve (C)). Experimentally, see Figure 4b (curves (D) and (E)), where the caffeine concentration rose steadily until reaching a plateau around the 11th cycle. The total amount of material necessary for the process until the 11th cycle was 476.1 mL of water and 88.19 g of coffee silverskin. The loss of water was similar for each cycle, as shown in Table 1, at around 26 mL.

At the 11th cycle, the extract had a volume of 175 mL, with a caffeine content of 0.835 g as measured with the spectrophotometer and 0.748 g given by UPLC analysis (around three times less than the predicted maximum value). The comparison between curves (C), (D), and (E) in Figure 4 leads us to conclude that the caffeine’s solubility at 25 °C is not the true limiting parameter since the observed limitation corresponds to a solubility of caffeine three times lower than the calculated value. A “salting-out” of caffeine was observed, probably due to co-extracted compounds such as sugars and minerals [47]. This means that the solubility of 9 wt% of pure caffeine in pure water at 60 °C could never be obtained in the extraction of coffee silverskin, because of the presence of other compounds.

The final extract, after 4 cycles without water compensation, contained 0.293 g of caffeine in 72.5 mL (4.04 mg/mL), compared to the 0.748 g measured by spectrophotometer and 0.835 g measured by UPLC (4.25–4.75 mg/mL) in 175 mL after 11 cycles with water compensation, depending on the measurement method. The loss of water during the process was 130 mL after 4 cycles without water compensation and 300 mL after 11 cycles with water compensation.

The caffeine/water loss ratio (*w*/*w*) without water compensation was 0.0022, while that with water compensation was 0.0028. Without water compensation, more than 22 cycles would be necessary to obtain the same quantities of caffeine as in the extract from 11 cycles with water compensation. To perform 22 cycles without water compensation, 700 mL of water would be necessary, in comparison to 460 mL of water with water compensation. Therefore, the process with water compensation seems to be the most adequate. A functional workflow can be obtained by keeping 10 or 11 cycles without adding unnecessary cycles. An improvement would be to find a way to enhance the caffeine solubility limit in the presence of co-extracts, but this option needs further research.

In the present contribution, around 2.1 g of caffeine was obtained for each 100 g of silverskin. This is higher than the values reported by Panusa et al. [48] (ranging from 0.3 to 0.4 g/100 g) and Bessada et al. [49] (ranging from 0.7 to 1.2 g/100 g) using water-based and hydroethanolic (1/1) dynamic maceration methods, respectively. Additionally, in a study conducted by Martuscelli and colleagues, the amount of caffeine extracted reached 1.7 g/100 g of silverskin using a 70% methanol mixture [50]. Although the higher contents obtained within this study may be linked to its higher efficiency, another potential explanation could be related to the variation in the initial content of caffeine in the raw materials due to agro-genetic factors.

### 2.3. Second Extraction of the Biomass

After extraction, one of the products obtained was a water-insoluble fiber fraction, exhausted from most of the soluble compounds. This exhausted silverskin was then subjected to a second extraction to evaluate the residual caffeine content. The results are compiled in Table 2 and compared to the amounts of caffeine extracted from the silverskin during the process.

For extractions with and without water compensation, an increase in the remaining caffeine in the exhausted biomass alongside the number of cycles was observed, with a maximum of 1.138 g per 200 mL of extract after 5 cycles without water compensation and 0.922 g per 200 mL of extract after 15 cycles with water compensation, when measured with a spectrophotometer. Hypothetically, a certain amount of caffeine could have been redeposited from the concentrated extract solution on the insoluble fibers. To obtain the maximum of amount of caffeine and insoluble fibers with a minimal content of caffeine, a second extraction with pure water is advisable for this process.

### 2.4. Thermogravimetric (TGA) Characterization of the Insoluble Fibers

**Figure 5:**
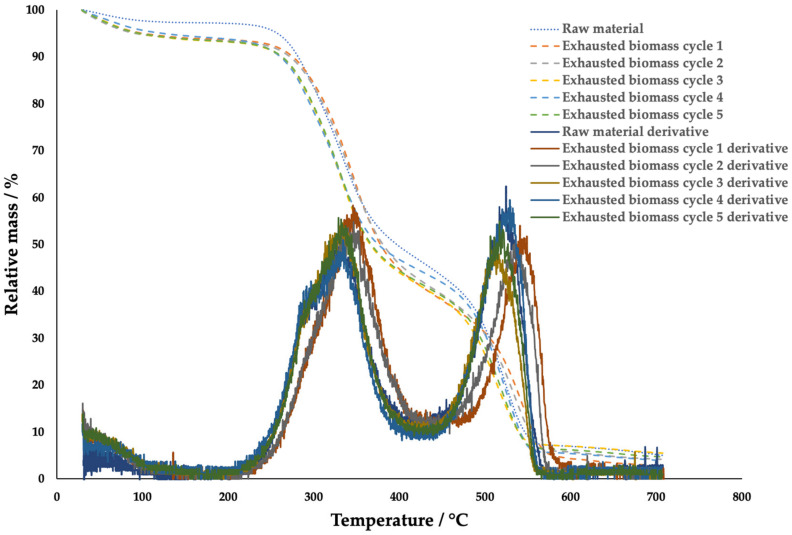
Thermogravimetric analysis of coffee silverskin and exhausted coffee silverskin from each cycle of the first type of extraction.

Insoluble fibers can be an issue for the process, considering their possible capture of caffeine after a certain number of cycles. Therefore, a verification of the composition changes of the biomass in different cycles was performed using thermogravimetric analysis. The analyses were conducted on the raw and exhausted biomass from the extraction without water compensation (see Figure 5), as the degradation of specific compounds can be observed by thermogravimetry [51,52]. The first degradation, taking place from room temperature to 150 °C, came from the evaporation of water and the volatile compounds bound to it. The second one, happening between 250 and 500 °C, was associated with the degradation of most compounds, such as the polyphenols, the oils, and some of the polysaccharides. The last one, until 710 °C, was the degradation of the remaining polysaccharide fractions. In the end, the only compounds left were ashes and minerals.

A difference between the raw materials and the exhausted ones was visible after the first and at the beginning of the second degradation stage. It can be concluded that, during the cycles, small water-soluble and mainly volatile molecules were extracted, while the main parts of the insoluble lignin and cellulose were not significantly affected.

### 2.5. Schematic Representation of the Process with Water Compensation and Potential Silverskin Valorizations

**Figure 6:**
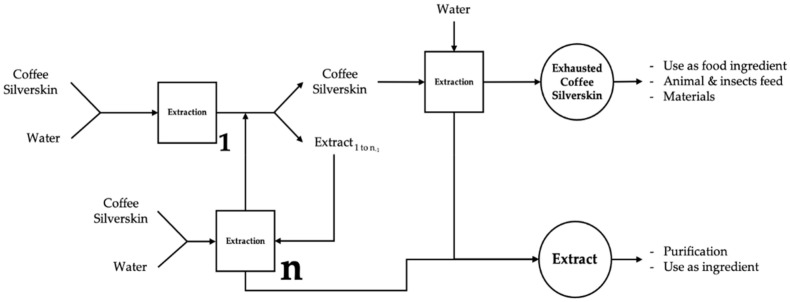
Designed process for coffee silverskin valorization with water compensation.

A schematic representation of the process is reported in Figure 6. The first extraction was carried out with water as a solvent and with silverskin. Afterwards, the exhausted silverskin was separated from the extract by straining and left to dry. The extract volume was then weighed and compensated to the original mass by the addition of water, to be used as a solvent for the next extraction on a new biomass. The number of cycles conducted should be 10–11. The final extract was obtained after all extraction cycles and a final straining. The dried exhausted biomass was then subjected to a second extraction with pure water and dried again.

The exhausted coffee silverskin is rich in insoluble fibers and polysaccharides, with a rich scent and color. The obtained fibers, containing none to a small amount of caffeine, have very low toxicity to animals and humans, and they are a good starting material to obtain lignin, cellulose, and related constituents and derivatives. This material could be used as an ingredient in the food or feed industries, e.g., as texturing agents [53], flour/starch [39], and flavoring or coloring agents. Other innovative uses for this product could be considered, e.g., the manufacturing of imitation leather [54].

The extract containing an interesting amount of caffeine could be used as it is, with few modifications, or as a base for the isolation of specific compounds.

The main caffeine beverages consumed are filter coffees, espressos, and energy drinks. Their caffeine contents are roughly the same, with 80–90 mg of caffeine in one drink [32]. Due to the significant difference in volume between espresso and the other two, its caffeine concentration is 1.33 mg/mL which is 3 to 4 times higher than in filter coffee and energy drinks (0.45 mg/mL and 0.32 mg/mL, respectively) [32]. The extraction process proposed here led to interesting results in terms of caffeine extracted compared to popular beverages (see Table 3). After one cycle, the caffeine concentration of the extract was 3 times greater than in filter coffee, and after eleven cycles the caffeine concentration was 11 times higher. Cycles can be useful to obtain a highly concentrated aqueous extract. One cycle of extraction could also be achieved using a simple adapted coffee machine.

This extract could be used as an ingredient for caffeinated and natural beverages, or as a concentrated water-dilutable beverage, to save storage, packaging, and costs. Any application should be associated with a proper and adapted preservation process to protect against biological growth and contamination.

Dichloromethane is still used today for its selectivity towards caffein from raw coffee beans at low temperatures extraction, despite its safety and sustainability concerns [55,56]. It could be advantageous to perform liquid–liquid extractions with the extract from the proposed process. Since the partition coefficient of caffeine between water and dichloromethane is highly in favor of the organic phase, this would lead to a dichloromethane phase highly concentrated in caffeine. A higher concentration of caffeine in the extract leads to a lower quantity of dichloromethane being required to extract the pure caffeine. It also seems worth mentioning that preliminary tests were conducted with water and ethyl acetate. However, the results were not satisfactory, because of the unfavorable partition coefficient of caffeine between water and ethyl acetate [42].

Pure, natural caffeine is a valuable chemical that can be used in different industries, such as in the food industry, in nutraceuticals [57,58], in cosmetics, or in pharmacy. According to a recent study, even the photovoltaics industry could use it [59].

## 3. Materials and Methods

### 3.1. Materials

Coffee silverskin from Arabica and Robusta was provided by the company Rehorik (Regensburg, Germany). The material used came without transformations, from the first crack of coffee roasting. The roasting was usually carried out with a quantity of beans varying from 25 to 40 kg and an average time of 30–40 min. It should be noted that each bean batch had its own characteristics.

### 3.2. Standard Extraction from Silverskin and Water-Insoluble Fibers

For the extractions, the coffee silverskin was weighed and placed in an Erlenmeyer flask with 200 mL of distilled water, inside a water bath with magnetic stirring, and heated to 60 °C. The temperature was regulated using an electronic thermometer linked to the water bath. After extraction, the mixture was passed through a strainer, and the exhausted material was pressed to remove excess water. The amount of water left was then measured to evaluate the water loss. The exhausted coffee silverskin was dried at 50 °C for 48 h.

Kinetics experiments with different ratios of biomaterial to water were conducted to observe the evolution of extraction efficiency depending on the mass-to-solvent ratio and time. The ratios tested were 1/100, 1/50, and 1/25. The kinetics experiments were carried out for 30 min at 60 °C, with a sampling at 0, 15, and 30 min.

Two types of extraction cycles were performed to concentrate the aqueous extract.

The first type of extraction cycle was carried out at a 1/25 ratio for 30 min. After each extraction, the exhausted biomass was strained, and a new one was added to start a new cycle, for a total of 5 cycles.

The second type of extraction cycle was conducted for 15 min at a ratio of 1/25. After straining the exhausted biomass, the water lost during the process was compensated by an equivalent amount of distilled water, and a fresh biomass was introduced to start a new cycle. In total, 15 cycles were performed.

An additional extraction was carried out with selected exhausted coffee silverskin at 60 °C, for 15 min, at a ratio of 1/25.

### 3.3. Calculations for Hypothetical Number of Cycles

Calculations to find the hypothetical number of cycles required to reach an optimum were carried out using a model introduced by Huber et al. in 2022 [60]. The model (see Equation (1)) was designed to calculate the number of possible cycles, with the help of UV–visible spectrophotometry measurements, before reaching an optimum for the extraction without adding water. The equation variables are the mass of caffeine in mg (m)**,** the number of cycles (n), the absorbance (A), the dilution of the extract for measurement (d), the molar extinction coefficient (ε), the thickness of the illuminated layer (l), the initial volume at the beginning of a cycle (V_i_), and the aqueous volume lost during the extraction (V_L_).

m(n) = ((n × A × d)/(ε × l)) × (V_I_ − (n × V_L_)) (1)

To determine the theoretical effect of the water loss, the established model was simplified to take into account water compensation at each cycle. For this, the term describing the water loss has to be removed, and the equation becomes

m(n) = (n × ((A × d)/(ε × l)) × V_I_) (2)

### 3.4. UV–Visible Spectrophotometric Determination of Caffeine Quantities Obtained by Water Extraction from Silverskin

Liquid samples were analyzed to determine the caffeine quantity through UV–visible spectrophotometry, using a PerkinElmer lambda19 (Dodgau, Germany) spectrophotometer; 200 µL aliquots of extract were taken and diluted adequately. First, a scan between 200 and 800 nm was performed for each sample, and the absorbance of the peak at 272 nm was determined.

### 3.5. UPLC UV–Visible Determination of Caffeine Quantities Obtained by Water Extraction from Silverskin

To analyze the caffeine contents, a Waters UPLC Acquity H class plus system with a PDA detector and an Acquity UPLC BEH C18-Column (1.7 μm, 50 mm × 2.1 mm) was used. The standard used was the 99.7% caffeine standard from Thermo Scientific. A calibration curve was prepared within a concentration range from 0 to 0.1 mg/mL.

The duration of the run was 12 min, at a constant elution flow of 8 mL/min. The mobile phase was made up of water with 0.1% formic acid (A) and acetonitrile (B). The elution gradient was selected as follows: from 0 to 4 min, 2% (B); from 4 to 6 min, 30% (B); from 6 to 6.50 min, 98% (B); from 6.50 to 8 min, 98% (B); from 8 to 8.50 min, 2% (B); then, the gradient remained at 2% (B) until the end of the run at 12 min.

All samples were injected at a volume of 2 µL. The chromatograms were registered at 273 nm and analyzed with Empower 3.0 software. The retention time of caffeine was 4.75 min. All analyses were performed in triplicate, and the obtained results were expressed as average values.

### 3.6. Thermogravimetric Analysis of the Water-Insoluble Fibers

Insoluble fibers were analyzed by thermogravimetric analysis with the TGA-7 from PerkinElmer. Measurements were conducted under synthetic air and nitrogen (80/20) at a flow rate of 25 mL/min. The temperature range studied was between 50 and 700 °C, at a heating rate of 10 °C/min.

## 4. Conclusions

In the present contribution, a process to extract caffeine using multi-cycle extraction, where the water content is compensated and the material renewed for each cycle, was investigated. Limiting the number of cycles to 11 allowed us to reach a plateau without unnecessary waste material. This study also revealed the value of the model proposed by Huber et al. to calculate the number of extraction cycles, and it proved its effectiveness when using water as a solvent. The proposed process resulted in two products: one liquid aqueous extract, with a high caffeine concentration of 4.25 mg/mL, and one exhausted solid composed of caffeine-rich insoluble fibers. These products have various potential applications. For instance, the extract could be used as a natural ingredient for beverages, or for the isolation of caffeine. The exhausted biomass could be used to produce materials, or as an ingredient in food or feed.

Bearing in mind that the proposed approach was conducted at a laboratory scale, its application at a larger scale holds tremendous potential from an industrial point of view. Hence, future research should focus on the scaling-up of the process, monitoring all potential factors that may influence the extraction efficiency. Another concern, which seems to be of pivotal importance, is the safety of the water used throughout the process. Although it is known as a natural, safe, and edible solvent, water should be thoroughly tested at all stages of the extraction process, in terms of both its physicochemical and microbiological properties, to ensure compliance with safety and quality standards prior to industrial use.

## Figures and Tables

**Table 1:** Comparison of water utilization: for extractions without the addition of water, the amount of water left after each cycle; for extractions with water compensation, the water used during the process.

Cycles without Water Addition		Cycles with Water Compensation	
Cycles	Water Left after Each Cycle (mL)	Cycles	Water Used during the Process (mL)
1	200.6	1	200.0
2	167.2	2	233.7
3	134.8	3	264.1
4	101.3	4	293.5
5	72.4	5	320.7
		6	346.2
		7	371.7
		8	397.0
		9	425.5
		10	449.9
		11	476.1
		12	500.3
		13	529.7
		14	551.9
		15	576.5

**Table 2:** Caffeine contents of the first versus the second extraction starting with fresh water for the same material, in order to evaluate the amount of remaining caffeine in the material. The amount of caffeine in grams is given for 200 mL of extract. Note that without the addition of water, the absolute volume is no longer 200 mL, but much less; therefore, the absolute amount of extracted caffeine is also lower. The caffeine values used are from the spectrophotometric measurements.

Cycles without Water Compensation				Cycles with Water Compensation			
Cycles	Caffeine Content in Extract (g/200 mL)	Caffeine Content in 2nd Extraction (g/200 mL)	Ratio	Cycles	Caffeine Content in Extract (g/200 mL)	Caffeine Content in 2nd Extraction (g/200 mL)	Ratio
1	0.156	0.066	2.4	1	0.146	n.d.	
2	0.324	0.090	3.6	5	0.575	0.122	4.7
3	0.534	0.134	4.0	10	0.869	0.163	5.3
4	0.810	0.148	5.5	15	0.922	0.170	5.4
5	1.138	0.175	6.5				

n.d. not defined.

**Table 3:** Comparison of the caffeine concentration, expressed in mg/mL, between popular caffeinated beverages and the extracts obtained with water solvation compensation. The reported caffeine contents for the extracts obtained in the present work were all measured with UPLC.

Products	Caffeine Content (mg)	Caffeine Concentration (mg/mL)	Equivalence in Filter Coffee Cups
Filter coffee (200 mL) ^1^	90	0.45	1
Espresso (60 mL) ^1^	80	1.33	3
Energy beverage (250 mL) ^1^	80	0.32	1
Extract after 1 cycle (170 mL)	171	1.03	3
Extract after 11 cycles (175 mL)	748	4.25	11

^1^ Numbers from an EFSA report on the risk of caffeine, 2015 [32].

## Data Availability

The original contributions presented in the study are included in the article, further inquiries can be directed to the corresponding author/s.
